# Comparison of Ultrasound-Guided Femoral Nerve Block and Intravenous Fentanyl For Analgesia During Positioning of Patients With Femur Fracture for Combined Spinal-Epidural Anesthesia: A Randomized Controlled Study

**DOI:** 10.7759/cureus.84201

**Published:** 2025-05-15

**Authors:** Hanford Bernnon Rajkumar, Abinaya Ramachandran, Nagalakshmi Palanisamy

**Affiliations:** 1 Anesthesiology, Chettinad Hospital and Research Institute, Kelambakkam, IND; 2 Anesthesiology, Pondicherry Institute of Medical Sciences, Pondicherry, IND; 3 Anesthesiology, Sri Manakula Vinayagar Medical College and Hospital, Pondicherry, IND

**Keywords:** epidural anaesthesia, femoral nerve block, femur and fracture, intravenous fentanyl, ultrasound-guided

## Abstract

Background

Central neuraxial blockade is the preferred anesthesia technique for femur fracture surgeries. However, positioning patients for neuraxial anesthesia can be challenging due to severe pain. Analgesic strategies, including nonsteroidal anti-inflammatory drugs (NSAIDs), opioids, non-opioid analgesics, and nerve blocks like lumbar plexus, fascia iliaca, three-in-one, and femoral nerve blocks (FNBs), are used to ease positioning. This study compared the analgesic efficacy of intravenous (IV) fentanyl and ultrasound-guided FNB using visual analogue scale (VAS) scores in patients with femur fractures undergoing combined spinal-epidural (CSE) anesthesia.

Methodology

Sixty-four American Society of Anesthesiologists (ASA) physical status I/II adult patients were randomized into two groups (n = 32). Group A received IV fentanyl (1 mcg/kg), while Group B received FNB with 20 mL of 0.25% bupivacaine under ultrasound guidance. Patients were positioned for CSE anesthesia. VAS scores and hemodynamic parameters were recorded at rest (V1), during movement (V2), and at 5 (V3), 10 (V4), and 15 (V5) minutes post-intervention.

Results

Demographics and hemodynamics were comparable between groups. Both groups showed significant VAS score reduction with no statistically significant difference at any time point (V1-V5, p > 0.05). The mean time to position patients was 4.25 ± 1.68 minutes in Group A and 3.63 ± 1.87 minutes in Group B (p = 0.166). The time for CSE administration was also similar (p = 0.861).

Conclusion

IV fentanyl is as effective as ultrasound-guided FNB for pain relief during patient positioning for CSE anesthesia in femur fracture surgeries. Ultimately, both methods are valuable analgesic strategies for facilitating patient positioning and improving the overall safety and comfort during the administration of central neuraxial anesthesia. The choice of intervention can be tailored based on individual patient characteristics, institutional protocols, and resource availability.

## Introduction

Femoral fractures are among the most frequently encountered long bone fractures in clinical practice, with an estimated incidence of 10 cases per one million population [1]. An essential component of successful surgical intervention is effective anesthesia. Central neuraxial blockade, which includes spinal, epidural, or combined spinal-epidural (CSE) techniques, is widely preferred for lower limb orthopedic surgeries due to its favorable safety profile and multiple perioperative benefits. These include better intraoperative and postoperative analgesia, lower opioid requirements, reduced incidence of postoperative nausea and vomiting, and decreased risk of thromboembolic events. These benefits collectively contribute to faster postoperative recovery, reduced length of hospital stay, and early discharge [2].

However, despite its advantages, neuraxial anesthesia presents a unique set of challenges in patients with femur fractures. These patients typically experience severe pain due to the nature of the fracture and associated soft tissue injury. Any movement, particularly those involved in transferring the patient onto the operating table or positioning them in either the sitting or lateral decubitus posture required for neuraxial blockade, can substantially aggravate their pain. This heightened discomfort not only increases patient distress but can also make it difficult for the anesthesiologist to obtain optimal positioning for the safe and effective administration of neuraxial anesthesia. Inadequate positioning may necessitate multiple attempts, which increases the risk of complications such as dural puncture, post-dural puncture headache, and, in rare cases, neurologic injury.

To address these challenges and enhance patient comfort, various analgesic strategies are employed prior to positioning for CSE. Systemic analgesics such as acetaminophen, nonsteroidal anti-inflammatory drugs (NSAIDs), and opioids like tramadol, fentanyl, and remifentanil have shown efficacy in alleviating pain [3-6]. However, opioid use is frequently limited by dose-dependent side effects such as respiratory depression, sedation, and gastrointestinal disturbances. Consequently, regional nerve blocks have gained popularity as an effective alternative. Techniques such as the fascia iliaca compartment block, perivascular nerve group block, and femoral nerve block (FNB) offer localized pain relief by targeting the nerve supply of the anterior thigh and femur without the systemic effects of opioids [7-10].

In this study, we sought to assess the analgesic effectiveness of intravenous (IV) fentanyl at a dose of 1 mcg/kg compared to ultrasound-guided FNB using 20 mL of 0.25% bupivacaine in patients undergoing surgical fixation of femur fractures under CSE anesthesia. The primary outcome was the assessment of pain using the visual analogue scale (VAS) during patient positioning. Secondary objectives included monitoring hemodynamic parameters before, during, and after the intervention, evaluating the ease and time required for positioning, and the duration of CSE administration.

## Materials and methods

Study design and ethical approval

This study was designed as a prospective, randomized, comparative clinical trial and conducted at Pondicherry Institute of Medical Sciences, Pondicherry, India. Ethical clearance was obtained from the Institutional Ethics Committee of Pondicherry Institute of Medical Sciences (Ref: IEC/RC/19/111), and the study was registered with the Clinical Trial Registry of India (CTRI/2022/01/039798). All participants provided written informed consent prior to enrollment, in accordance with ethical standards.

Sample size calculation

The required sample size was calculated based on the primary outcome variable, which was the difference in mean VAS score five minutes after intervention. This calculation was based on data from a study by Buddhi and Gupta [11], where the mean VAS score after a femoral nerve block (FNB) was 7.21 ± 1.38 and after intravenous (IV) fentanyl was 8.03 ± 0.93. Assuming an 80% power and a 5% level of significance, a minimum of 32 patients was required per group.

Study population

The study included a total of 64 adult patients aged between 16 and 80 years, who were classified as American Society of Anesthesiologists (ASA) physical status I or II. These patients were scheduled for surgical fixation of femur fractures. Exclusion criteria were established to maintain patient safety and included the presence of head injury or altered sensorium with a low Glasgow Coma Scale (GCS) score, bilateral femur or pelvic fractures, known allergy to local anesthetics, bleeding disorders, or an international normalized ratio (INR) above the normal reference range.

Preoperative preparation

All patients underwent standard preoperative fasting for at least six hours. Upon arrival in the preoperative holding area, pain was assessed using the VAS, with scores recorded both at rest (V1) and during movement while transferring onto the operating table (V2). Once in the operating room, baseline parameters including heart rate (HR), mean arterial pressure (MAP), and peripheral oxygen saturation (SpO₂) were measured. An 18-gauge IV cannula was placed, and IV fluid therapy was initiated using Ringer's lactate at a rate of 15 mL/kg/hour.

Randomization and group allocation

**Figure 1:**
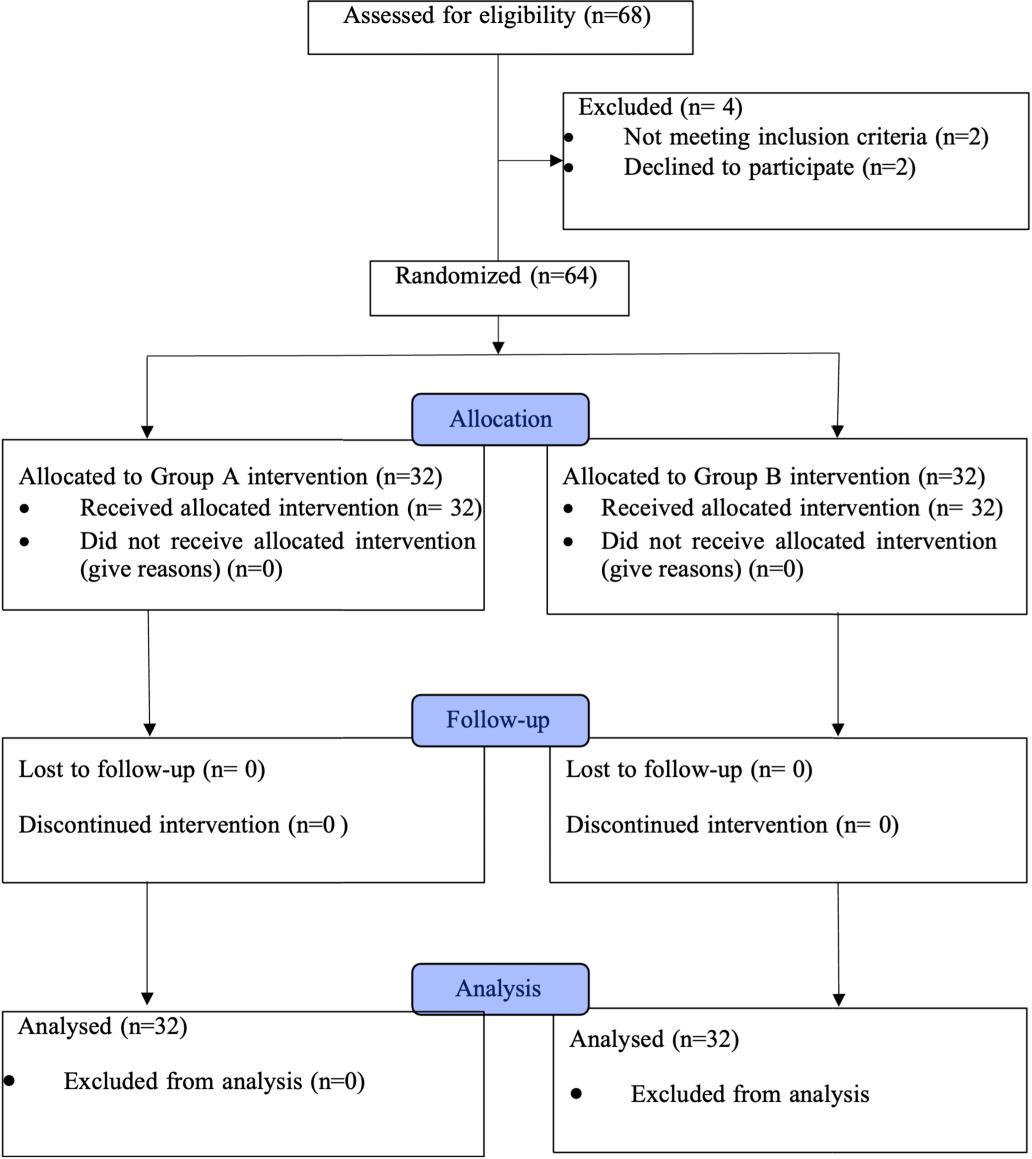
CONSORT flow diagram

Participants were randomized into two groups (32 each), based on a computer-generated block randomization chart. Group A received IV fentanyl at a dose of 1 µg/kg, administered 15 minutes prior to positioning for CSE anesthesia. Group B underwent an ultrasound-guided FNB using 20 mL of 0.25% bupivacaine, also administered 15 minutes before the patients were positioned for the CSE procedure. The CONSORT diagram is provided in Figure 1.

Femoral nerve block technique

For patients in Group B, the FNB was performed under ultrasound guidance. A high-frequency linear probe (6-13 MHz) was used to identify the femoral nerve, located lateral to the femoral artery and beneath the fascia iliaca in the inguinal region. Following strict aseptic precautions and local infiltration with 1% lidocaine, a 22-gauge needle was inserted using the in-plane technique, and the local anesthetic solution was deposited around the nerve after confirming the correct location.

Pain and hemodynamic monitoring

After administration of the analgesic intervention, pain was reassessed using the VAS at five-minute intervals over the following 15 minutes. These were recorded as V3 at 5 minutes, V4 at 10 minutes, and V5 at 15 minutes. Simultaneously, continuous monitoring of hemodynamic parameters such as HR, MAP, and SpO₂ was maintained throughout the intervention and positioning period, up to the successful administration of the CSE anesthesia.

Positioning and anesthesia administration

At the end of the 15-minute analgesia window, patients were assisted into the appropriate position for CSE anesthesia. The time required for positioning was recorded, followed by the time taken to complete the CSE procedure. In cases where patients reported a VAS score greater than 4 following the initial analgesic intervention, additional doses of IV fentanyl (0.5 µg/kg) were administered at five-minute intervals until the VAS score dropped below 4, ensuring optimal patient comfort during positioning.

Statistical analysis

All data were analyzed using IBM SPSS Statistics for Windows, Version 20.0 (Released 2011; IBM Corp., Armonk, NY, United States). Continuous variables were summarized as mean ± SD or median with IQR, while categorical data were expressed as proportions and analyzed using the chi-square (χ²) test. Hemodynamic trends were evaluated using unpaired t-tests and validated through repeated measures ANOVA. Since VAS scores represent ordinal data, differences between groups at each time point were assessed using the Mann-Whitney U test. A p-value less than or equal to 0.05 was considered statistically significant in all analyses.

## Results

Demographic variables, including age, height, and weight, demonstrated no statistically significant differences between Group A and Group B. Age distributions, gender distribution, anthropometric measures, and ASA classifications (e.g., ASA I vs. ASA II) were comparable, confirming successful randomization (Table 1).

**Table 1:** Demographic variables of the study participants (N = 32) p < 0.05 is considered statistically significant. Independent t-test.

Demographic variables	Group A (mean ± SD)	Group B (mean ± SD)	p-value
Age (years)	51.09 ± 16.99	52.63 ± 21.37	0.759
Height (cm)	165.97 ± 7.27	169.91 ± 5.87	0.020
Weight (kg)	70.72 ± 13.26	74.88 ± 11.94	0.193

**Figure 2:**
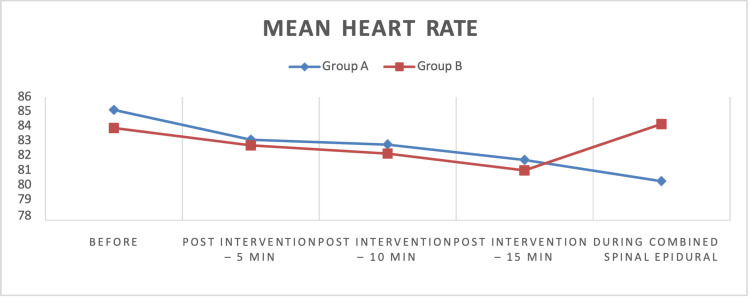
Line diagram showing the mean comparison of heart rate between the two groups before intervention, post-intervention (5, 10, and 15 minutes), and during combined spinal-epidural anesthesia

VAS scores for pain at rest, during movement, and post-intervention (5, 10, and 15 minutes) were comparable between groups at all time points. Median VAS scores, as depicted in the box plot (Figure 2), showed overlapping IQRs and similar central tendencies, with no statistically significant differences reported. This consistency indicates equivalent analgesic efficacy between Group A and Group B interventions across all measured intervals (Table 2).

**Table 2:** Comparison of VAS scores between the two groups at rest, during movement, and post-intervention (5, 10, and 15 minutes) p < 0.05 is considered statistically significant. Mann-Whitney U test.

VAS	Group A		Group B		Mann-Whitney “U”	Z	Sig (p-value)
	Median (Q3-Q1)	Mean rank	Median (Q3-Q1)	Mean rank			
At rest (V1)	7 (8-6)	31.13	7 (8-6)	33.88	468.0	0.605	0.545
During movement (V2)	8 (10-7)	31.39	8.5 (9.75-8)	33.61	476.5	0.488	0.625
Post-intervention (5 minutes; V3)	6 (8-5)	33.48	6 (7-5)	31.52	480.5	0.431	0.667
Post-intervention (10 minutes; V4)	6.50 (7-4)	36.38	5 (6-4)	28.63	388.0	1.691	0.091
Post-intervention (15 minutes; V5)	5 (6.75-4)	36.33	4 (5-4)	28.67	389.5	1.675	0.094

Positioning for neuraxial anesthesia took 4.25 ± 1.68 minutes in Group A versus 3.63 ± 1.87 minutes in Group B (p > 0.166), reflecting no significant difference. CSE anesthesia performance times were also similar: 5.91 ± 1.55 minutes for Group A and 6.02 ± 2.57 minutes for Group B (p = 0.861). These results suggest comparable procedural efficiency between the two groups. Mean HR trends were analogous between groups. Before intervention, Group A had a mean HR of 86 beats per minute (bpm), compared to 81 bpm in Group B. Post-intervention, HR decreased gradually in both groups: 5 minutes (Group A, 85 bpm; Group B, 80 bpm), 10 minutes (Group A, 84 bpm; Group B, 79 bpm), and 15 minutes (Group A, 83 bpm; Group B, 78 bpm). During CSE anesthesia, HR stabilized at 82 bpm in Group A and 78 bpm in Group B, with no statistically significant intergroup differences observed (Figure 2).

**Figure 3:**
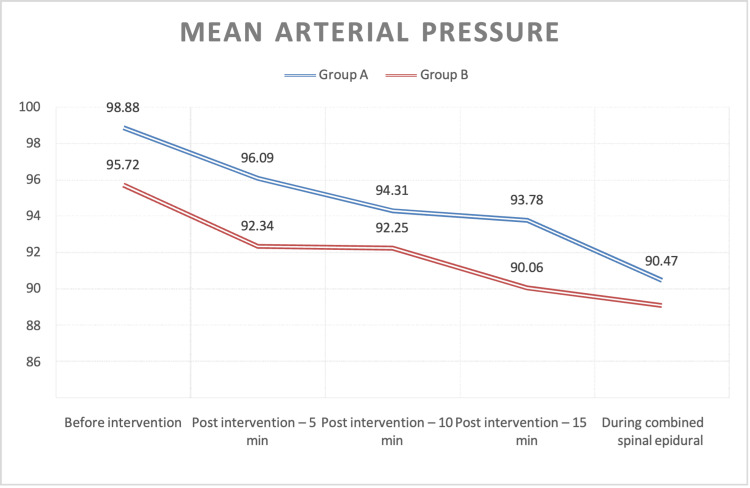
Line diagram showing the mean comparison of mean arterial pressure (MAP) between the two groups before intervention, post-intervention (5, 10, and 15 minutes), and during combined spinal-epidural anesthesia

MAP followed a declining trend post-intervention in both groups. Group A started at 100 mmHg before intervention, decreasing to 98.88 mmHg (5 minutes), 96.09 mmHg (10 minutes), 94.31 mmHg (15 minutes), and 92.34 mmHg (during CSE anesthesia). Group B began at 95.72 mmHg, declining to 93.78 mmHg (5 minutes), 92.25 mmHg (10 minutes), 90.06 mmHg (15 minutes), and 90.47 mmHg (during CSE anesthesia). Despite numerical variations, statistical analysis revealed no significant differences between groups at any time point (Figure 3).

## Discussion

With an incidence of 10 per 1,000,000, femur fracture repair remains one of the most frequently performed orthopedic procedures. Surgery is the preferred treatment, with central neuraxial blockade often favored due to its advantages. However, the intense pain and immobility following trauma hinder proper patient positioning for neuraxial blockade [12,13]. Inadequate pain relief during positioning not only compromises patient comfort and satisfaction but can also contribute to the development of chronic pain syndromes. Suboptimal positioning may result in multiple attempts to secure neuraxial catheters, even in otherwise straightforward cases.

Various pharmacological and interventional techniques are employed to alleviate pain and facilitate optimal positioning. Among these, the comparative efficacy of femoral nerve block (FNB) versus intravenous (IV) analgesia remains debated. While some studies highlight the superiority of FNB, others argue for the utility of IV agents such as fentanyl, ketamine, midazolam, and dexmedetomidine [14]. Jadon et al. [15] compared IV fentanyl (1 µg/kg) and FNB using 20 mL of 1.5% lidocaine with adrenaline for positioning patients for spinal anesthesia. Their results indicated significantly lower VAS scores and reduced time to complete spinal anesthesia in the FNB group, with better quality of positioning.

Another study demonstrated that administering FNB with 15 mL of 1.5% lignocaine five minutes before spinal anesthesia was superior to IV fentanyl at 3 µg/kg. The FNB group had significantly lower VAS scores (p < 0.001), though some in the IV group experienced oxygen desaturation below 90% and required additional fentanyl [16]. These findings may be explained by the faster onset of lidocaine and the inadequate five-minute interval allowed for fentanyl to reach peak plasma levels.

Our study compared the analgesic effects of IV fentanyl (1 µg/kg) with ultrasound-guided FNB using 20 mL of 0.25% bupivacaine, administered 15 minutes prior to positioning for CSE anesthesia. Pain was assessed using VAS at rest (V1), during movement (V2), and at 5, 10, and 15 minutes post-intervention (V3-V5). VAS scores in both groups significantly decreased after intervention, with median scores falling from 7 to 5 in the IV fentanyl group and from 7 to 4 in the FNB group. However, the difference between groups was not statistically significant (p = 0.094), suggesting comparable efficacy.

Our results align with Lamaroon et al. [17] who found no significant difference in Numeric Rating Scale (NRS) scores between groups receiving FNB (20 mL of 0.5% bupivacaine + 10 mL saline) or IV fentanyl (0.5 µg/kg every five minutes until NRS ≤ 4). However, unlike their study, none of our patients required additional fentanyl doses, thus avoiding associated adverse effects like sedation, pruritus, and nausea. We used the time taken for patient positioning and completion of CSE as indicators of patient comfort and procedural ease. 

Contrastingly, George et al. [18] reported significantly lower VAS scores (1.97 ± 0.56 vs. 2.87 ± 0.35) and shorter positioning time (178.33 ± 32.73 s vs. 210.17 ± 14.05 s) in the FNB group (20 mL of 1.5% lignocaine) compared to IV fentanyl (1 µg/kg). Yun et al. [19] compared FNB with 1.5% lignocaine and adrenaline against 0.5 µg/kg IV fentanyl in femoral fracture surgeries. While IV fentanyl provided adequate analgesia, it often required repeat doses, and the positioning quality was slightly inferior in the FNB group, possibly due to underdosing. Buddhi and Gupta [11] compared IV fentanyl (2 µg/kg) with an ultrasound-guided FNB using a combination of 0.375% bupivacaine and 0.5% lignocaine. The FNB group had significantly lower VAS scores and fewer side effects than the IV group, where some patients experienced drowsiness due to the higher fentanyl dose.

Our study also monitored hemodynamic parameters, including HR and MAP, at baseline and 5, 10, and 15 minutes post-intervention, as well as during the CSE procedure. The mean HRs before and after intervention showed no significant difference between groups, remaining consistent across all time points. These findings are consistent with Kumar et al. [20] who reported no significant difference in HR at baseline or during positioning between patients receiving ultrasound-guided FNB (15 mL of 1% lignocaine) and those given 1 µg/kg of IV fentanyl.

Regarding MAP, both groups experienced a decline from baseline post-intervention, but differences between groups were not statistically significant at any time point. Baseline MAPs were 98.88 ± 14.05 (Group A) and 95.72 ± 12.10 (Group B), decreasing steadily post-intervention and during CSE. Jadon et al. [15] also noted a significant MAP reduction in the IV fentanyl group compared to FNB using 20 mL of 1.5% lignocaine with adrenaline. This reduction was attributed to fentanyl and lignocaine-adrenaline combinations. Additionally, two fentanyl patients exhibited higher sedation scores. Gupta and Kamath [21] compared ultrasound-guided FNB and fascia iliaca block (FIB) using 30 mL of 0.25% bupivacaine in proximal femur fracture surgeries. Both provided effective pain relief without significant hemodynamic changes.

A notable limitation of our study was the absence of blinding, potentially introducing observer bias. Future studies could incorporate blinding to enhance internal validity. Additionally, evaluating patient satisfaction during positioning could provide further insights into analgesic efficacy. We suggest FNB as a preferred approach, particularly for individuals at risk of opioid problems, due to its similar efficacy and possibility for fewer opioid-related adverse effects. Patient satisfaction measures should be included in future studies to improve analgesic tactics for the patient group. 

## Conclusions

Our study demonstrates that both IV fentanyl and ultrasound-guided FNB provide effective analgesia for patient positioning during neuraxial anesthesia in femur fracture surgeries, with no significant differences in pain scores, procedural times, or hemodynamic stability. Given the comparable efficacy and potential for fewer opioid-related side effects, we recommend considering FNB as a preferred technique, especially in patients at risk of opioid complications. Future research should incorporate blinding and patient satisfaction metrics to further refine analgesic strategies for this patient population.
